# Community Pharmacy Recruitment for Practice-Based Research: Challenges and Lessons Learned

**DOI:** 10.3390/pharmacy11040121

**Published:** 2023-07-22

**Authors:** Jessica Roller, Anna Pfeiffer, Courtney Humphries, Chloe Richard, Jon Easter, Stefanie Ferreri, Melanie Livet

**Affiliations:** UNC Eshelman School of Pharmacy, University of North Carolina at Chapel Hill, Chapel Hill, NC 27599, USA

**Keywords:** community pharmacy services, pharmacy research, evidence-based pharmacy practice, value-based health care

## Abstract

To support the successful integration of community pharmacies into value-based care models, research on the feasibility and effectiveness of novel pharmacist-provided patient care services is needed. The UNC Eshelman School of Pharmacy, supported by the National Association of Chain Drug Stores (NACDS) Foundation, designed the Community-based Valued-driven Care Initiative (CVCI) to (1) identify effective value-based patient care interventions that could be provided by community pharmacists, (2) implement and evaluate the feasibility of the selected patient care interventions, and (3) develop resources and create collaborative sustainability opportunities. The purpose of this manuscript is to describe recruitment strategies for CVCI and share lessons learned. The project team identified pharmacies for recruitment through a mixed data analysis followed by a “fit” evaluation. A total of 42 pharmacy organizations were identified for recruitment, 24 were successfully contacted, and 9 signed on to the project. During recruitment, pharmacies cited concerns regarding the financial sustainability of implementing and delivering the patient care services, challenges with staffing and infrastructure, and pharmacists’ comfort level. To foster participation, it was vital to have leadership buy-in, clear benefits from implementation, and assured sustainability beyond the research period.

## 1. Introduction

The United States healthcare system lags behind its peers in terms of care quality, efficiency and patient outcomes. The U.S. consistently ranks last in health outcomes but spends the most at 16.8% of the gross domestic product, far exceeding peer countries [1]. In an effort to address this, the U.S. healthcare landscape is shifting toward value-based (or outcomes-based) care, where providers receive payment based on patient outcomes instead of the volume of services provided. Value-based care focuses on improving patient health, reducing the burden of chronic disease, and using evidence-based strategies to help patients live healthier lives [2]. Pharmacists are recognized medication management experts and have demonstrated improvements in patient outcomes across a variety of chronic diseases such as diabetes, dyslipidemia, and hypertension [3,4]. Therefore, important opportunities exist to expand the provision of value-based care into community pharmacies for purposes of achieving better healthcare quality, efficiency and outcomes.

Community pharmacies are one of the most accessible healthcare access points with almost 90% of people in the United States living within 5 miles of a community pharmacy [5]. Many patients see their pharmacist more frequently than their primary care physician [6]. Furthermore, the availability of primary care physicians continues to decline particularly in rural areas, with a projected primary care physician shortage in the United States of 17,800 to 48,000 by 2034 [7,8]. Community pharmacists are in a prime position to fill this access gap and some already are through the provision of enhanced patient care services such as wellness screenings, comprehensive medication management, point of care testing, and disease management [9]. However, this practice is not widespread, and research is limited on strategies to improve the implementation and availability of more patient care services at community pharmacies. While community pharmacists generally have positive attitudes toward research, barriers persist including lack of experience participating in research, not being aware of research opportunities and time constraints—all of which work to prevent their participation and ultimately create barriers to patients’ access to care [10].

### Community Pharmacy Value-Based Care Initiative

To support the successful integration of community pharmacies into value-based care models, research on the feasibility and effectiveness of novel pharmacist-provided patient care services is needed. In support of this goal, the UNC Eshelman School of Pharmacy, supported by the National Association of Chain Drug Stores (NACDS) Foundation, designed the Community-based Valued-driven Care Initiative (CVCI). This initiative had three aims. The first was to identify effective patient care interventions that could be provided by community pharmacists to improve care access, quality, and efficiency. The second aim was to implement and evaluate the feasibility of the selected patient care interventions in community pharmacies. The third aim was to develop and disseminate resources to educate other healthcare providers around the United States, as well as create collaborative sustainability opportunities by connecting community-pharmacy-based care intervention programs with other community healthcare partners as a foundational component of a sustainability strategy.

The selected patient care interventions included a comprehensive diabetes program, a comprehensive cardiovascular disease program, and a behavioral health support program for depression and anxiety. After developing the interventions, including the creation of educational toolkits, the team set out to recruit community pharmacies for participation in the study. National and regional chain pharmacies, grocery store pharmacies, as well as independent pharmacies across the country were contacted for recruitment. Recruited pharmacies participated in the study for a period of 15 months consisting of a pre-implementation period (3 months) and implementation period (12 months). The purpose of this manuscript is to describe recruitment strategies and share lessons learned.

## 2. Recruitment Strategy

Recruitment occurred from October 2020 through to June 2022. Throughout this time, the project team, which was composed of implementation science researchers and a community pharmacist consultant, met weekly to review potential pharmacy participants and discuss challenges to recruitment and implications to the initiative timeline.

### 2.1. Identifying Pharmacies to Recruit

A list of potential pharmacy organizations was developed using a two-step approach. Possible research partners were first identified through the use of mixed data including an environmental scan and informal literature review, a review of applicable state prescriptive and collaborate practice agreement regulations, and stakeholder interviews and expert committee input. This list was then refined using a number of criteria to evaluate the potential fit based on the information gathered, including pharmacy type (chain, supermarket or independent), state(s) where the organization operated, current patient care programs offered, culture of the organization, leadership, current capacity, and relationships. Following the selection of the three patient care services to be implemented, the initial recruitment list was revisited. Based on discussions of the initial fit information collected above and of the three available patient care services, the project team prioritized pharmacy organizations identified as being innovative, with a culture and leadership that promoted advancing patient care services and pharmacy practice. A recruitment target of three to five pharmacy organizations per patient care intervention was set.

### 2.2. Contacting Eligible Pharmacies for Recruitment

An initial email containing information about the research initiative and an invitation to participate was sent to each pharmacy organization using the contact information obtained during the pharmacy identification process. If no response was received within 7 days, a second email was sent. If no response was received after the second email, pharmacies were contacted via phone.

Initial meetings were scheduled with each pharmacy organization’s point of contact and research team members to describe the research initiative and assess key considerations for “fit” using a checklist [11]. The purpose of the fit checklist was to ensure foundational compatibility between the pharmacy setting and the selected patient care service. Fit considerations included alignment with: (1) needs and metrics, (2) organizational resources and capabilities, (3) organizational priorities and culture, (4) financial long-term sustainability, and (5) intervention feasibility with the applicable regulatory environment (Appendix A). Pharmacies deemed a good fit were scheduled for a second meeting to provide information on patient care interventions and determine pharmacy capabilities and technology supports. All meetings were conducted using videoconferencing software.

Using the combined results from the initial pharmacy identification process and the fit assessment, the project team considered the following variables in determining eligibility for participation: sufficient patient population for the targeted interventions, synergy with other patient care services, capability of staff and software, alignment with pharmacy organization priorities, support of leadership, and allowance through state legislation for the intervention. Pharmacies identified as meeting all necessary criteria were provided with a letter of commitment which outlined research initiative expectations. Upon receipt of the signed commitment letter, the pharmacy organization was established as a research initiative participant.

## 3. Recruitment Results

The landscape analysis identified 42 potential community pharmacy organizations to contact. These included 20 chains, 21 independents, and 1 health system outpatient community-based pharmacy (Table 1).

Of the 42 pharmacy organizations identified in the landscape analysis, 24 were successfully contacted. Meetings were held with 22 of the organizations and 9 signed the commitment letter (Table 2).

Most of the pharmacy organizations that joined the research initiative were independents with single or multiple locations. Two regional pharmacy chains agreed to participate (Table 3).

## 4. Community Pharmacy Recruitment: Lessons Learned

Successfully recruiting community pharmacy organizations for participation in the research initiative proved challenging. During the recruitment process, the project team noted pharmacy concerns regarding the financial sustainability of implementing and delivering the patient care services, challenges with staffing and infrastructure, and pharmacists’ comfort level in providing care specifically for the behavioral health intervention. To foster pharmacy participation, the project team also found that it was vital to have leadership buy-in, clear benefits from implementation, and assured sustainability beyond the research period.

### 4.1. Pharmacies Want to Know that Patient Care Services Implemented Can Be Sustained beyond the Conclusion of the Research Initiative

Throughout the recruitment period, pharmacy organizations questioned the sustainability of patient care services following the completion of the research initiative. Many pharmacy organizations stated that there was not a viable path to sustainability for the patient care services without a financial model. This is a commonly described barrier to implementation of novel patient care services in community pharmacies [12,13]. While there are a handful of established financial models supporting quality-driven patient care services [14,15], they are currently not common or widespread. While many stakeholders, including payers, may believe that community pharmacies can and should offer more patient care services [16], most models do not comprehensively provide coverage for pharmacy-based services on a widespread basis. Community pharmacy participation in research could produce data to support future financial models, which ultimately support improved patient access to care and health outcomes. It is also interesting to note that among pharmacists who have implemented new services, financial factors are not the only motivational factors [15,17]. Therefore, focusing recruitment efforts on pharmacies whose mission and values prioritize patient care may help promote success in conducting community-pharmacy-based research.

### 4.2. Pharmacies Must Be Ready to Adopt and Implement New Patient Care Services

The project team built in a planning (or readiness) phase for participating pharmacy research sites to prepare for implementation through pharmacy education, trainings and adequate staffing, identification of patient populations who may benefit from these services, development of the missing infrastructure necessary for implementation (e.g., data systems, space), integration of these services into existing patient care workflows, and working with leadership to ensure that these services were a priority for the pharmacy organization. This phase was guided by an evidence-informed readiness assessment and building process described elsewhere [18,19]. During these planning efforts, pharmacy organizations expressed specific concerns over their ability to deliver new patient care services. For example, implementation of medication therapy management services is challenged with dedicating space for private conversations with clients [20]. The pharmacy organizations in this study also specifically identified adequate physical space and staff support as barriers to their participation in the study. Regarding physical space, barring renovations of the building, solutions are limited. However, pharmacies that provide immunizations may already have an area that is private and separated from foot traffic. To overcome the challenges of limited staff, the project team encouraged pharmacy organizations to consider how they could use non-pharmacist support staff, such as pharmacy technicians and clerks, to assist with the non-clinical aspects of the patient care workflow, such as educating patients about the enhanced care services during checkout and scheduling patient appointments. In addition, the pharmacies that serve as preceptor sites for pharmacy students and residents were advised to consider how learners could be used to support the delivery of new care services. The impact of pharmacy student implementation of patient care services in the community setting has been previously described as being a beneficial experience for students, pharmacies, and patients [21,22,23]. Pharmacy sites were educated regarding the use of medication synchronization and applying the appointment-based model (ABM) to further overcome staffing challenges. Medication synchronization and the ABM have been found to not only increase medication adherence but also streamline dispensing workflow to allow more time for direct patient care services [24,25,26,27].

### 4.3. Behavioral Health Services Were More Challenging for Pharmacy Staff to Adopt and Deliver than Chronic Diseases Management Services

The behavioral health intervention proved to be the most challenging for recruitment. Community pharmacists can make a significant impact in behavioral health care around areas like screening and linkage to care, in addition to addressing adverse drug reactions, medication and disease state monitoring, and medication counseling [28,29,30]. However, many pharmacy organizations reported that they were not equipped to discuss anxiety and depression with their patients based on a lack of experience. This aligns with research to date, as previous studies found that pharmacists are more comfortable with hypertension and diabetes management than they are with counseling on behavioral health medications [31]. This was also reflected in our conversations with pharmacy organizations; it was easier to recruit pharmacy organizations to participate in the cardiovascular disease and diabetes interventions than behavioral health. Stigma toward mental health disorders contributes to hesitancy to provide behavioral health counseling [32]. One reason for stigma and a lack of comfortability could be a lack of experience using clinical knowledge around psychotropic medications, which can be resolved by offering targeted education and training [33]. An example of targeted training which has improved health professionals’ comfort around mental health conditions is the Mental Health First Aid (MHFA) training [34,35]. MHFA training has been found to increase confidence around mental health crises and decrease stigma and social distance [35]. Improving comfortability with mental health conditions could increase staff buy-in for behavioral health interventions. Before implementing a behavioral health patient care intervention in community pharmacies, it is important to provide adequate education and training on psychotropic medications and the provision of mental health care.

### 4.4. Organizational Leadership Buy-in Was Necessary for Pharmacy Participation, Assuming Alignment with Frontline Pharmacy Sites

Regardless of individual pharmacist interest, without the support of organizational leadership, it was challenging to recruit pharmacy organizations. Leadership buy-in is a commonly cited facilitator for the implementation of new patient care services, as support from the top can either make or break organizational change efforts [36,37]. It is also important to note that as barriers arise during the implementation of new services, it is usually organizational leadership that can address those barriers [38]. As a result, the project team focused recruitment efforts on pharmacy organizations whose mission and values were expressly aligned with the aims of the CVCI as this increased the likelihood of leadership buy-in. By describing how this research initiative’s patient care services matched the pharmacy organizations’ vision and could help address gaps in care for their patient populations, organizational support increased.

### 4.5. The Effects of COVID-19 Negatively Impacted Pharmacy Recruitment Efforts

Because recruitment started during the pandemic, the increased burden placed on community pharmacies at that time affected recruitment efforts. The Public Readiness and Emergency Preparedness (PREP) Act expanded the role of community pharmacists nationwide by authorizing and relying on them to provide COVID-19 vaccines, testing, and treatments, including for children and adolescents [39]. This abrupt shift in focus meant that pharmacies often did not have the capacity to take on additional patient care services at the time the research was conducted. Similarly to the rest of the healthcare ecosystem, new public health initiatives were also accompanied by increased burnout from increased prescription volume and implementation of new immunization and testing services [40]. Additionally, the safe social distancing and isolation recommendations from the Centers for Disease Control and Prevention (CDC) may have limited patient foot traffic through pharmacies [41]. This presented challenges with patient contact during the normal clinical services workflow as many patients began using the drive thru windows to maintain social distancing. While some of these physical challenges may have been resolved with a transition to telehealth, not all pharmacies had the feasibility for that transition. Common barriers experienced when implementing telepharmacy services during the COVID-19 pandemic included lack of space for private conversations with patients, inadequate pharmacy technology, and lack of training and experience to date for pharmacists conducting telepharmacy services [42]. Overall, pharmacists and pharmacies were limited, and many could not commit to providing additional patient care services. As a result, the project team delayed recruitment efforts until the impact of the pandemic seemed to have subsided enough for pharmacies to consider participation.

### 4.6. Final Thoughts and Considerations for Future Recruitment Efforts

During the CVCI recruitment period, the project team overcame hurdles to successfully recruit 21 community pharmacy sites from 9 pharmacy organizations. The challenges identified include concerns regarding sustainability, infrastructure, staff level of comfort with behavioral health services, organizational support, and the impacts of the COVID-19 pandemic. To address these challenges, we found that it was key to focus on pharmacy organizations that are patient care “innovators” with organizational priorities expressly aligned with the target interventions and mission of the research effort. Innovators, as defined by Rogers’ Diffusion of Innovations Theory, are those who are willing to take risks and act as change agents in their field [43]. These innovator pharmacies are typically independents and supermarket pharmacies [44,45]. Possible reasons for this include direct pharmacy owner involvement, the comparative ease of implementation in fewer pharmacy sites, and the overall mission of innovator supermarket pharmacies in contributing to patients’ whole health [44]. This does not mean that chain and mass-market pharmacies do not have missions supporting patient care and are not vital to the broad delivery of patient care services, but it is important to note that independent and supermarket pharmacies historically have been the early adopters, whereas most chain and mass-market pharmacies have been late adopters [44,45].

Successfully recruiting community pharmacies to adopt value-based patient care services hinges on meeting the needs of the pharmacies and reducing barriers prior to implementation. Community pharmacists have already demonstrated that they are valuable public health professionals [46,47,48]. By incorporating additional value-based patient care services into their patient care workflow, community pharmacies have the potential to further contribute to improvements in the health of their communities.

## 5. Conclusions

Community pharmacy participation in research is vital to inform the advancement of accessible, community-pharmacy-based patient care interventions that improve healthcare access, efficiency and outcomes. Researchers should consider how to overcome potential barriers to participation prior to the start of recruitment efforts. By ensuring that community pharmacies’ concerns are addressed up front, researchers may have greater success in recruiting community pharmacies for research participation.

## Figures and Tables

**Table 1:** Community pharmacy landscape analysis summary.

Pharmacy Type	*n* (%)
Chain (national and regional)	20 (48%)
Independent (single and multiple locations)	21 (50%)
Health system	1 (2%)
Total	42 (100%)

**Table 2:** Community pharmacy recruitment engagement.

Intervention	Contacted	Initial Meeting	CVCI Participants
Diabetes	4	4	2
Cardiovascular Disease	4	4	3
Behavioral Health	13	11	4
Total	24	22	9

**Table 3:** Community pharmacy demographics by patient care intervention.

Intervention	Pharmacy Type	State	Number of Sites
Diabetes	Regional grocery chain	VA, NC	5
	Independent	NC	1
Cardiovascular Disease	Regional grocery chain	PA	2
	Independent	IA	5
	Independent	IA	1
Behavioral Health	Independent	MO	3
	Independent	IA	1
	Independent	IA	2
	Independent	NC	1

## Data Availability

The data presented in this study are available on request from the corresponding author. The data are not publicly available as the study is ongoing and data is not yet available.

## Appendix A. Fit Checklist

Purpose: To quantify the level of readiness to each fit consideration category and identify areas of improvement.

Use: To be filled out by the project team member during/after discussion with site. Each subcomponent should be marked as “present”, “absent”, “needs improvement”, or “N/A”.

**Fit Consideration**	**Subcomponent**
1. Alignment with needs and metrics	- Has a way to understand patients’ needs - Patient population has sufficient need for this intervention - Interventions available to address patient needs - Clear metric to measure intervention success - Synergistic with other community services - Likely favorable perception of intervention from patients
2. Alignment with organizational resources and capabilities	- Technology and data capabilities to identify patients in need - Technology and data capabilities to document patient encounter - Technology and data capabilities to collect pertinent patient data needed for service - Technology and data capabilities for monitoring and reporting program metrics - Ability to communicate effectively with patients - Adequate staff to dedicate to additional intervention - Staff comfortable delivering selected intervention - Partnerships/existing relationships in place for referral destination - Dedicated clinical space - Coverage of initial operational expense
3. Alignment with organizational priorities and culture	- Clear alignment of the service with organizational priorities - Clear alignment of the service with organizational culture - Clear alignment of the service with organizational philosophy of patient care - Leadership supports the trial of the service - Leadership is dedicated to removing barriers and embracing necessary organizational changes to make service successful
4. Alignment with reimbursement mechanisms for long-term sustainability	- Direct billing mechanism present - Identified other potential reimbursement mechanisms for intervention - Can articulate how service contributes to organization’s investment priorities
5. Alignment with regulatory environment	- State allows for full operationalization of intervention - Invention is in compliance with organization’s regulations
